# Phenotypic Evolution, Clinical Subtypes, and Independent Predictors of Long COVID: A Retrospective Cohort Study

**DOI:** 10.3390/biomedicines14081662

**Published:** 2026-07-24

**Authors:** Lanre Peter Daodu, Yogini Raste, Judith E. Allgrove, Francesca I. F. Arrigoni, Reem Kayyali

**Affiliations:** 1 Faculty of Health, Sciences, Social Care and Education, Kingston University, London KT1 2EE, UK; f.arrigoni@kingston.ac.uk (F.I.F.A.); reem.kayyali@kingston.ac.uk (R.K.); 2 Chest/Respiratory Department, Croydon University Hospital, London CR7 7YE, UK; yoginiraste@nhs.net; 3 School of Allied Health and Exercise Sciences, Faculty of Health, Environment and Medical Sciences, Bournemouth University, Bournemouth BH8 8GP, UK; jallgrove@bournemouth.ac.uk

**Keywords:** long COVID, post-acute sequelae of SARS-CoV-2, phenotypic evolution, clinical subtypes, latent class analysis

## Abstract

**Background:** Post-acute sequelae of COVID-19 (PASC), commonly known as long COVID, affects an estimated 10–30% of SARS-CoV-2 non-hospitalised and 50–70% of hospitalised survivors. This condition remains clinically heterogeneous, and the specific mechanisms driving the transition from acute infection to chronic sequelae remain poorly understood. We assessed independent risk factors, tracked the evolution of clinical features, defined distinct symptom-based phenotypes, and assessed the impact of different pandemic waves on the likelihood of developing long COVID in hospitalised survivors. **Methods:** We conducted a single-centre, retrospective cohort study at a university hospital in London. The population comprised 627 adults hospitalised with acute COVID-19 between February 2020 and December 2022. Baseline characteristics and outcomes were compared between long COVID and resolved cases using appropriate statistical tests for continuous and categorical variables. Multivariable logistic regression identified risk factors. McNemar’s test quantified the phenotypic shift from admission to follow-up. Latent Class Analysis (LCA) identified clinical subtypes based on symptom clusters. **Results:** Of 627 patients, 252 (40.2%) met long COVID criteria. Comorbidity burden was the strongest predictor; patients with a single condition had a 4-fold increase in odds (aOR 4.62, 95% CI 2.36–9.04). The ORs were also significantly elevated among patients with 2 (aOR 3.26), 3 (aOR 2.68), or 4 or more (aOR 3.24) comorbidities. Older age (aOR 1.04), acute disease severity (measured by length of hospital stay) (aOR 1.27 per log-day) and elevated admission fibrinogen (aOR 1.21 per g/L) were significant predictors. Temporal analysis revealed a precipitous decline in risk from Wild-type/Alpha (>50%) to Delta/Omicron (<21%). We observed a distinct phenotypic shift: while acute respiratory inflammation resolved, systemic fatigue increased fourfold (7.0% to 32.4%), and memory difficulties emerged in the post-acute phase. LCA identified two phenotypes: fatigue-dominant and multisystem phenotypes. **Conclusions:** Long COVID is a multifactorial syndrome driven by host susceptibility, acute severity, and persistent coagulopathy. Clinical management should move beyond a monolithic approach and favour phenotype-specific strategies.

## 1. Introduction

The acute phase of the SARS-CoV-2 pandemic has precipitated a secondary, chronic public health challenge. This condition is characterised by the persistence of debilitating symptoms long after viral clearance. The World Health Organisation (WHO) refers to this as post-COVID-19 condition, or long COVID [1]. The occurrence is estimated to be 10–30% among non-hospitalised individuals [2], 50–70% among hospitalised individuals [3,4], and 10–12% among vaccinated individuals [5,6]. This condition imposes a substantial burden on global healthcare systems, workforce productivity, and quality of life. As the pandemic transitions into an endemic phase, the focus of clinical research must shift from acute survival to understanding the determinants of chronic morbidity, particularly in vulnerable populations who require hospitalisation.

Despite the accumulation of epidemiological data, the condition remains clinically heterogeneous, and the specific mechanisms driving the transition from acute infection to chronic sequelae remain incompletely understood [7]. While broad demographic predictors such as advanced age and multimorbidity are well-established [8,9], the biological drivers of this persistence are less clear. Prevailing hypotheses suggest a complex, multifactorial aetiology involving viral persistence in tissue reservoirs, immune dysregulation and autoimmunity, autonomic nervous system dysfunction, and gut dysbiosis [10,11]. Additionally, emerging evidence points to “immunothrombosis”—sustained endothelial activation and coagulopathy—as another potential contributing pathological mechanism [10]. The clinical presentation of long COVID is distinct from the acute phase. While acute COVID-19 is primarily a respiratory syndrome characterised by fever and dyspnoea, anecdotal evidence suggests a phenotypic shift toward a systemic syndrome dominated by fatigue and neurocognitive dysfunction in the post-acute phase [12,13]. However, few studies have utilised paired data to quantitatively map this evolution within the same patient cohort. Additionally, long COVID is likely not a monolithic entity but rather a collection of distinct clinical subtypes (phenotypes). Unravelling this heterogeneity is essential for precision medicine, yet few studies have employed probabilistic modelling to define these sub-phenotypes based on symptom clusters [14].

Existing research has been constrained by significant methodological limitations. Large-scale electronic health record studies often rely on diagnostic coding or self-reported surveys, which, while powerful in sample size, frequently lack granular biological data. Crucially, retrospective cohorts usually exclude patients with incomplete laboratory records, introducing selection bias and preventing the analysis of biomarkers such as fibrinogen that may be non-randomly missing [15]. Furthermore, few studies have utilised paired longitudinal data to quantitatively map the phenotypic evolution of symptoms within the same patient cohort, leaving a gap in our understanding of how the disease metamorphoses from acute respiratory distress to the systemic neurocognitive syndromes observed in the post-acute phase [12,15].

In this study, we employed a retrospective cohort design to longitudinally track patients from acute hospital admission through to the post-acute follow-up period. This approach enables the assessment of temporal sequences between baseline exposures and subsequent outcomes while controlling for confounding variables such as viral variant waves. Using a deeply phenotyped dataset, we aimed to determine independent predictors of long COVID in hospitalised patients, assess how the clinical presentation evolves from the acute phase (COVID) to the post-acute phase (post-COVID), and quantify the phenotypic shift from acute respiratory symptoms to chronic systemic or neurocognitive sequelae. In addition, we assessed whether the heterogeneity of long COVID can be categorised into distinct clinical phenotypes (subtypes) using probabilistic modelling, and whether these phenotypes correspond to varying levels of symptom burden. Lastly, we evaluated how the risk of developing long COVID changed across the pandemic’s chronological waves, with a specific focus on comparing the pre-vaccination era (Wild-type/Alpha variants) with the post-vaccination era (Delta/Omicron variants).

## 2. Materials and Methods

### 2.1. Study Design and Setting

We conducted a single-centre retrospective observational cohort study at a university hospital in South London, United Kingdom. The study involved a review of electronic patient records (EPRs) and was conducted between June 2023 and June 2024. The cohort comprised patients hospitalised for acute COVID-19 at the hospital between February 2020 and December 2022. The findings were reported in accordance with the Strengthening the Reporting of Observational Studies in Epidemiology (STROBE) guidelines.

### 2.2. Participants and Eligibility

The study included adult patients (aged ≥ 18 years) with a laboratory-confirmed diagnosis of SARS-CoV-2 infection via reverse transcription-PCR or antigen testing who required hospitalisation. We excluded patients under 18 years of age, patients without confirmed COVID-19 test results, and patients who died during the acute phase of infection (defined operationally as in-hospital mortality during the index COVID-19 admission or death within 4 weeks of the initial positive test). This was done to avoid competing risks in the assessment of long-term sequelae, which requires patient survival up to the 12-week follow-up threshold.

### 2.3. Outcome Measures

The primary outcome was the development of long COVID (post-COVID-19 condition), defined according to WHO criteria as the persistence of symptoms for at least 12 weeks following initial infection [1]. Operationally, this was treated as a binary outcome, dichotomising the study population into those with persistent sequelae versus those with fully resolved infection. Classification was ascertained through a stepwise composite review. First, patients were identified via the ICD-10 code for post-COVID-19 condition (U09.9). Concurrently, manual abstraction of post-discharge clinical notes (e.g., respiratory follow-up clinic notes and available primary care records) was performed to track 14 core WHO-aligned symptoms across respiratory, systemic, neurocognitive, and musculoskeletal domains [1]. To distinguish long COVID from underlying comorbidities, symptoms were only included if they were documented as new-onset post-infection or a significant exacerbation of a baseline condition clinically attributed to COVID-19. Also, to control for age-related confounders, particularly in older patients with pre-existing conditions such as early-stage dementia, neurocognitive symptoms (e.g., *‘*brain fog*’* or memory difficulties) were only coded as post-acute sequelae if explicitly documented by a clinician as new-onset following the viral infection, or as an acute, uncharacteristic exacerbation of their baseline cognitive state. Patients were classified as ‘resolved’ if clinical notes explicitly confirmed full recovery or if there was no documented evidence of persistent sequelae > 12 weeks post-infection. Any ambiguous cases—particularly those concerning the attribution of symptoms to long COVID versus pre-existing conditions or alternative infections—were flagged and underwent clinical adjudication by the physician responsible for the patients. The secondary outcomes include identifying independent predictors of the disease condition, characterising the phenotypic evolution of the disease by quantifying changes in clinical presentation from the acute to the post-acute phase, and identifying distinct clinical phenotypes. We evaluated temporal dynamics to determine how the risk of developing long COVID evolved across the pandemic’s chronological phases; this involved stratifying patients by specific pandemic waves—Wild-type, Alpha, Delta, and Omicron—to assess the impact of changing viral variants on long-term outcomes.

### 2.4. Data Collection and Variable Definitions

Data extraction was performed using Castor Electronic Data Capture (EDC) software, v2024.2. Extracted variables included age, sex, ethnicity, body mass index (BMI), and smoking status. Extracted underlying health conditions included hypertension (HTN), diabetes mellitus (DM), cardiovascular diseases, respiratory conditions (asthma, chronic obstructive pulmonary disease (COPD), interstitial lung disease (ILD)), chronic kidney disease (CKD), neurological disorders, and malignancy. The extracted temporal metrics were the dates of the first positive test, admission, and discharge. In addition, acute infection symptoms at admission and post-acute sequelae (defined as symptoms persisting for at least 12 weeks after the initial infection) were extracted. Laboratory biomarkers included haematological indices; coagulation profiles (international normalised ratio [INR], activated partial thromboplastin time [APTT], fibrinogen, D-dimer); renal function and electrolytes (creatinine, urea, sodium); liver function (albumin, alanine aminotransferase [ALT]); and inflammatory markers (C-reactive protein [CRP], ferritin).

### 2.5. Statistical Analysis

Statistical analyses were performed using R Statistical Software, R version 4.6.1. All statistical tests were two-tailed, and a *p*-value of <0.05 was considered statistically significant.

### 2.6. Descriptive Statistics and Univariate Analysis

Baseline characteristics were compared between patients who developed long COVID and those with resolved infection. Continuous variables were assessed for normality using histograms and Q-Q plots (Supplementary Figure S1). Normally distributed variables were reported as means with standard deviations (SDs) and compared using the independent *t*-test. Non-normally distributed variables were reported as medians with interquartile ranges (IQRs) and compared using the Mann–Whitney U test. Categorical variables were reported as frequencies and percentages, with group differences assessed using Pearson’s Chi-square test or Fisher’s exact test. Clinical follow-up duration was calculated as the number of months from hospital discharge to the last documented clinical encounter or to the end of the retrospective chart review period.

### 2.7. Temporal and Trend Analysis

To evaluate the impact of changing viral variants on long COVID risk, patients were stratified into four pandemic waves based on the date of their first positive SARS-CoV-2 test and national UK genomic surveillance timelines [16,17]: Wave 1 (Wild-type: Feb 2020–Aug 2020), Wave 2 (Alpha: Sept 2020–May 2021), Wave 3 (Delta: June 2021–Nov 2021), and Wave 4 (Omicron: Dec 2021–Aug 2022). Differences in the prevalence of long COVID across these waves were assessed using Pearson’s chi-square test. An epidemic curve was constructed by aggregating cases into monthly intervals to visualise the distribution of outcomes over the study period.

### 2.8. Paired Symptom Analysis

To characterise the phenotypic shift from acute infection to post-acute sequelae, a paired analysis was conducted on clinical symptoms recorded at two distinct time points (admission vs. ≥12 weeks post-discharge) for the same study subjects. McNemar’s test was applied to paired categorical data to evaluate statistical differences in the prevalence of specific symptoms between the acute and post-acute phases.

### 2.9. Clinical Phenotyping via Latent Class Analysis

To address the clinical heterogeneity of long COVID and identify distinct pathophysiological sub-phenotypes, Latent class analysis (LCA) was performed on the subset of patients with confirmed post-acute sequelae. LCA was utilised as the gold-standard probabilistic method to group patients based on symptom patterns rather than traditional distance-based clustering [18,19]. Fourteen core symptoms spanning respiratory, neurocognitive, systemic, and musculoskeletal domains were entered into the model. Models specifying two to four latent classes were fitted using the poLCA package. The optimal number of classes was determined by minimising the Bayesian information criterion (BIC) and by assessing clinical interpretability. Following cluster identification, phenotypes were characterised and named based on their conditional symptom probabilities, and each patient was assigned to the cluster with the highest posterior probability.

### 2.10. Predictors of Phenotype Membership

To investigate whether distinct biological drivers predispose patients to specific symptom clusters, a subsequent logistic regression model was fitted using the LCA-derived phenotypes as the dependent variable. This analysis evaluated whether specific independent variables, such as admission fibrinogen levels, age, and acute length of hospital stay (LoS), were associated with membership in the “severe” or “multisystem” phenotype. Results are reported as odds ratios (ORs) with 95% confidence intervals (CIs).

### 2.11. Handling of Missing Data

Data completeness was assessed before analysis (Supplementary Table S1). Variables with excessive missingness (>50%), including d-dimer, ferritin, troponin T (TnT), and creatine kinase (CK), were excluded from the multivariable analysis to avoid introducing bias. For variables with partial missingness (<30%), MICE was employed. We utilised predictive mean matching (PMM) to account for the non-normal distribution of laboratory biomarkers. To ensure statistical stability given the proportion of missing data, 30 imputed datasets (m = 30) were generated. The outcome variable, long COVID status, was included in the imputation model to preserve associations but was not imputed.

### 2.12. Variable Selection and Engineering

Before regression analysis, variables were engineered for clinical relevance. The raw count of comorbidities (0–12) was categorised into five groups (0, 1, 2, 3, and ≥4) to detect non-linear risk patterns. For the laboratory biomarkers, a univariate screening approach was applied to the imputed data. Laboratory variables with *p*-values < 0.05 in the univariate analysis were included in the final model. Redundant variables measuring similar physiological functions, such as urea vs. creatinine, were screened to prevent multicollinearity, as they are often highly correlated; the most clinically standard marker was retained. The final multivariable logistic regression model included age, sex, ethnicity, BMI, comorbidities, and the selected laboratory biomarkers.

### 2.13. Multivariable Regression Analysis and Model Diagnostics

Multicollinearity was assessed using the variance inflation factor (VIF), with a threshold of <5 indicating no significant collinearity. Linearity assumptions for continuous variables, such as age and BMI, were visually verified using component-plus-residual (CPR) plots. A binary logistic regression model was fitted to identify independent risk factors for long COVID. The model included sociodemographic characteristics (age, sex, ethnicity), lifestyle (smoking status), clinical factors (comorbidities, BMI), and selected laboratory biomarkers. Results from the 30 imputed datasets were pooled using Rubin’s rules to generate valid standard errors and confidence intervals [20,21]. Results were reported as adjusted odds ratios (aORs) with 95% CIs.

### 2.14. Sensitivity Analysis

To verify the robustness of the multiple imputation strategy, a sensitivity analysis was conducted using Complete Case Analysis (CCA) [22,23]. CCA involved running the same multivariable logistic regression model on the original unimputed dataset, excluding any patients with missing values. The direction, magnitude, and significance of the ORs were compared between the primary (imputed) model and the sensitivity (complete case) model.

## 3. Results

### 3.1. Baseline Characteristics of the Study Population

**Figure 1:**
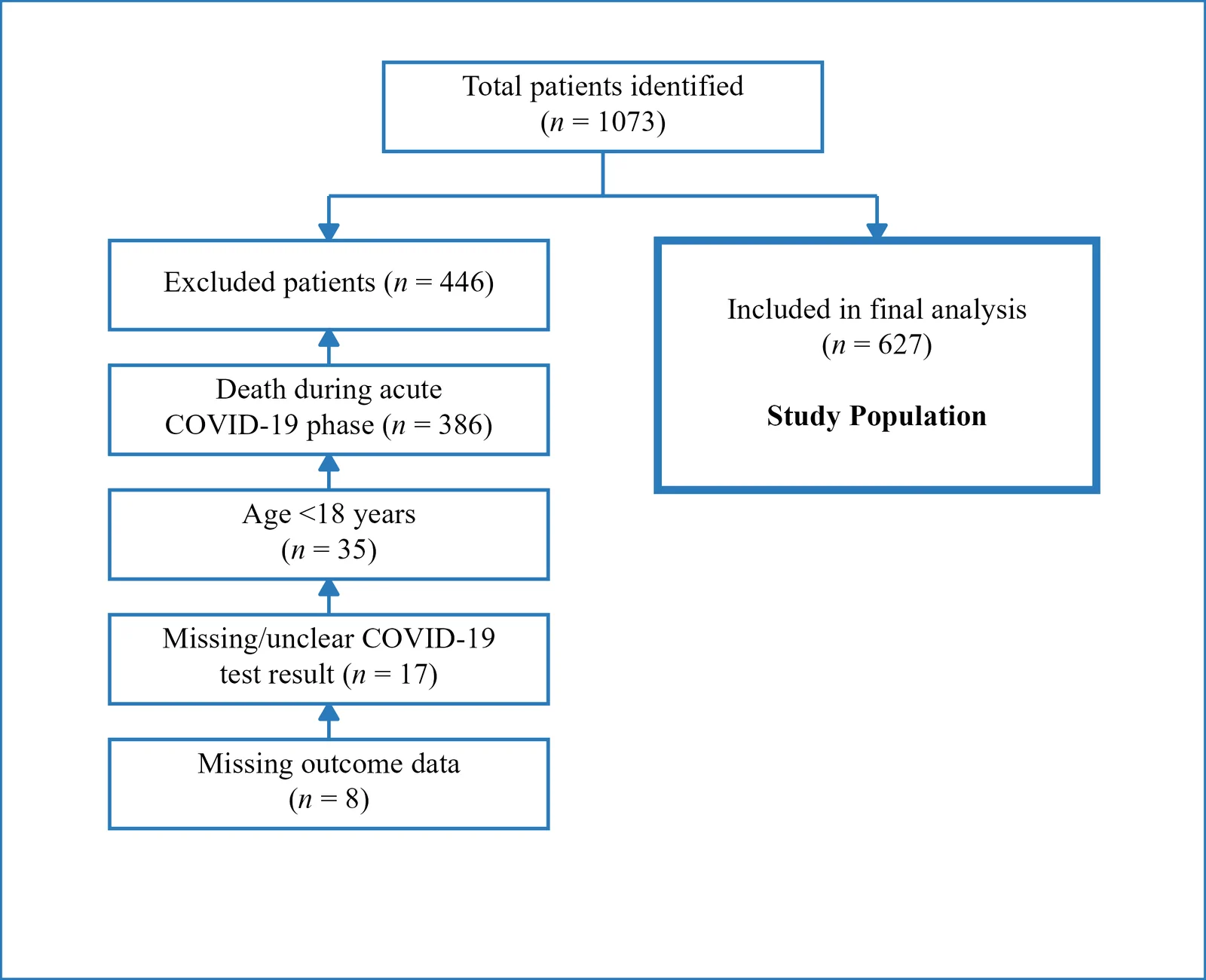
STROBE flow diagram illustrating the study participant selection process. The flowchart details the exclusion criteria applied to the initial pool of electronic patient records (*n* = 1073).

During the study period, an initial pool of 1073 EPRs was identified for screening. After applying predefined exclusion criteria—most notably the removal of 386 patients who died during the acute COVID-19 phase—a final analytical cohort of 627 adult patients was established (Figure 1).

A total of 627 patients hospitalised with acute COVID-19 were included in the analysis. Of these, 252 (40.2%) met the criteria for long COVID, while 375 (59.8%) experienced full symptom resolution. The baseline demographic, clinical, and laboratory characteristics are summarised in Table 1. Patients who developed long COVID were significantly older than those who recovered fully (mean age 69 ± 16 years vs. 53 ± 20 years; *p* < 0.001). There were no statistically significant differences between the groups regarding biological sex (*p* = 0.269), ethnicity (*p* = 0.738), or BMI (*p* = 0.290). Baseline underlying health condition was a strong differentiator between the two groups (*p* < 0.001). A clear gradient in comorbidity burden was observed: only 9.1% of patients in the long COVID group had no comorbidities, compared to 39% in the resolved group.

Nearly half (46%) of the long COVID patients had 4 or more comorbidities, compared with 25% in the resolved group. Smoking status showed a statistically significant difference between groups (*p* = 0.035), with a higher proportion of former smokers in the long COVID group (15% vs. 11%), though current smoking rates were similar (7.1% vs. 9.9%). The median LoS was significantly longer in the long COVID group compared to the resolved group (6.0 days [IQR 4.0–9.0] vs. 4.0 days [IQR 3.0–7.0]; *p* < 0.001). The median duration of clinical follow-up post-discharge for the entire cohort was 28.1 months (IQR: 16.4–29.3). When stratified by pandemic wave, follow-up duration was longest for the Wild-type/Alpha waves (median 29.0 months) and shortest for the Omicron wave (median 15.5 months).

Patients with long COVID exhibited significantly higher median levels of fibrinogen (5.70 g/L vs. 5.20 g/L; *p* < 0.001) and INR (1.10 vs. 1.00; *p* < 0.001), as well as elevated serum creatinine (81 µmol/L vs. 76 µmol/L; *p* = 0.001). Serum albumin levels were significantly lower in the long COVID group (*p* = 0.024). No statistically significant difference in serum sodium levels was observed (*p* = 0.057). A higher percentage of the long COVID patients were vaccinated (72.6%) compared to the resolved patients (56.0%).

### 3.2. Temporal Dynamics and Long COVID Prevalence

**Figure 2:**
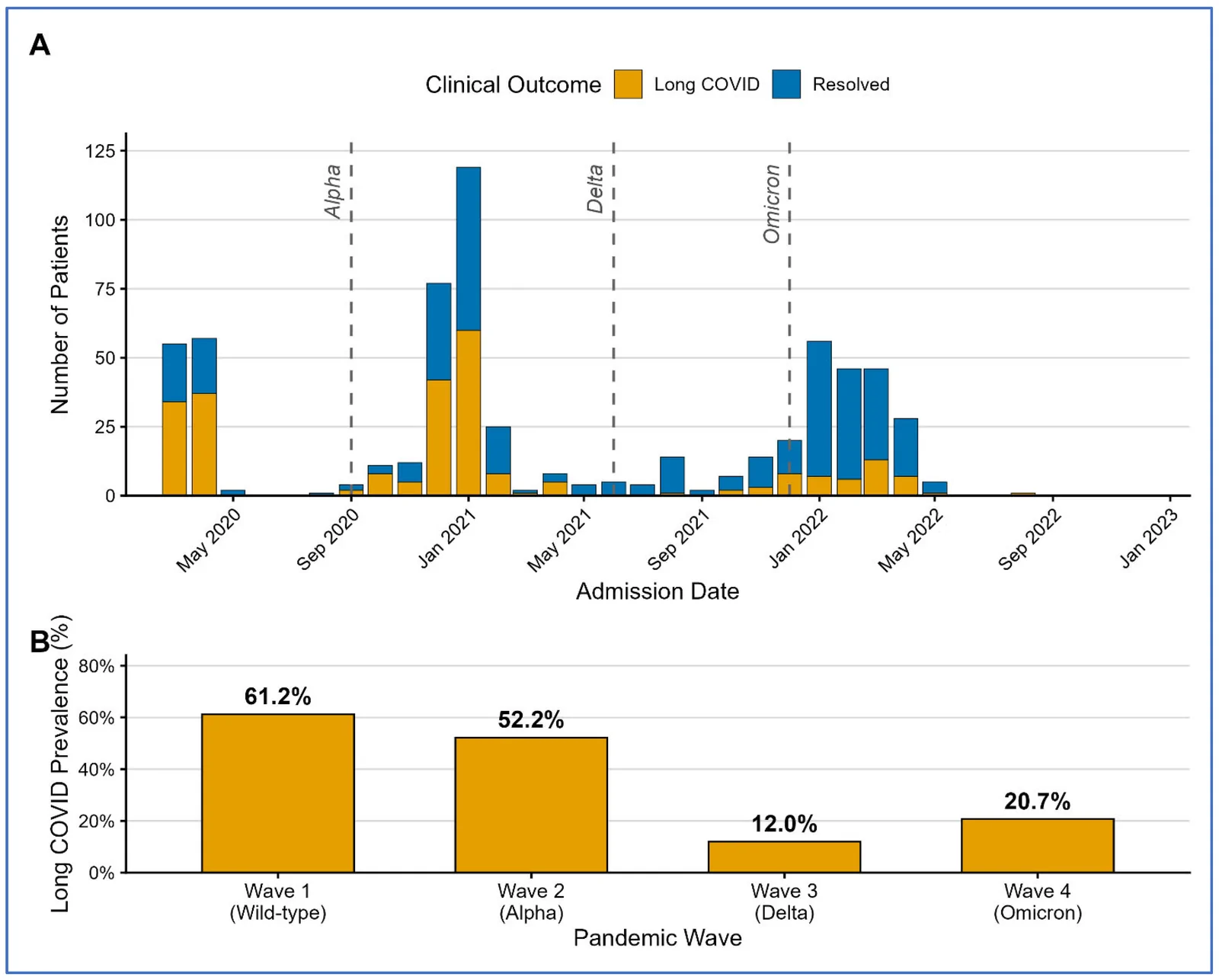
Temporal dynamics of acute COVID-19 hospital admissions and subsequent long COVID prevalence. (**A**) Epidemic curve illustrating the monthly distribution of hospital admissions for acute COVID-19 from February 2020 to December 2022, stratified by clinical outcome (Dark blue = Resolved, Dark goldenrod = Long COVID). Vertical dashed lines indicate the approximate onset of major SARS-CoV-2 variant waves in the UK. (**B**) Bar chart displaying the prevalence of long COVID stratified by chronological pandemic wave.

The epidemic curve (Figure 2A) showed a distinct shift in outcomes from February 2020 to December 2022. During the initial phases (Wild-type and Alpha waves), a substantial proportion of hospitalised patients developed long COVID. A significant temporal shift in risk was observed across the pandemic waves (*p* < 0.001). As illustrated in Figure 2B, the prevalence of post-acute sequelae dropped precipitously following the emergence of the Delta variant (12.0%) compared to the Wild-type (61.2%) and Alpha (52.2%) waves (*p* < 0.001). Although a slight increase in risk was observed during the Omicron wave (20.7%), it remained significantly lower than the rates observed during the first year of the pandemic.

### 3.3. Independent Predictors of Long COVID

Model assumptions were verified before interpretation. Multicollinearity was assessed using the VIF. All predictors showed VIF values < 2.5, indicating no significant collinearity (Supplementary Figure S2). Linearity assumptions for continuous variables (age, BMI, fibrinogen, and log-transformed LoS) were visually confirmed using component-plus-residual plots, which showed no significant deviation from linearity (Supplementary Figure S3).

**Figure 3:**
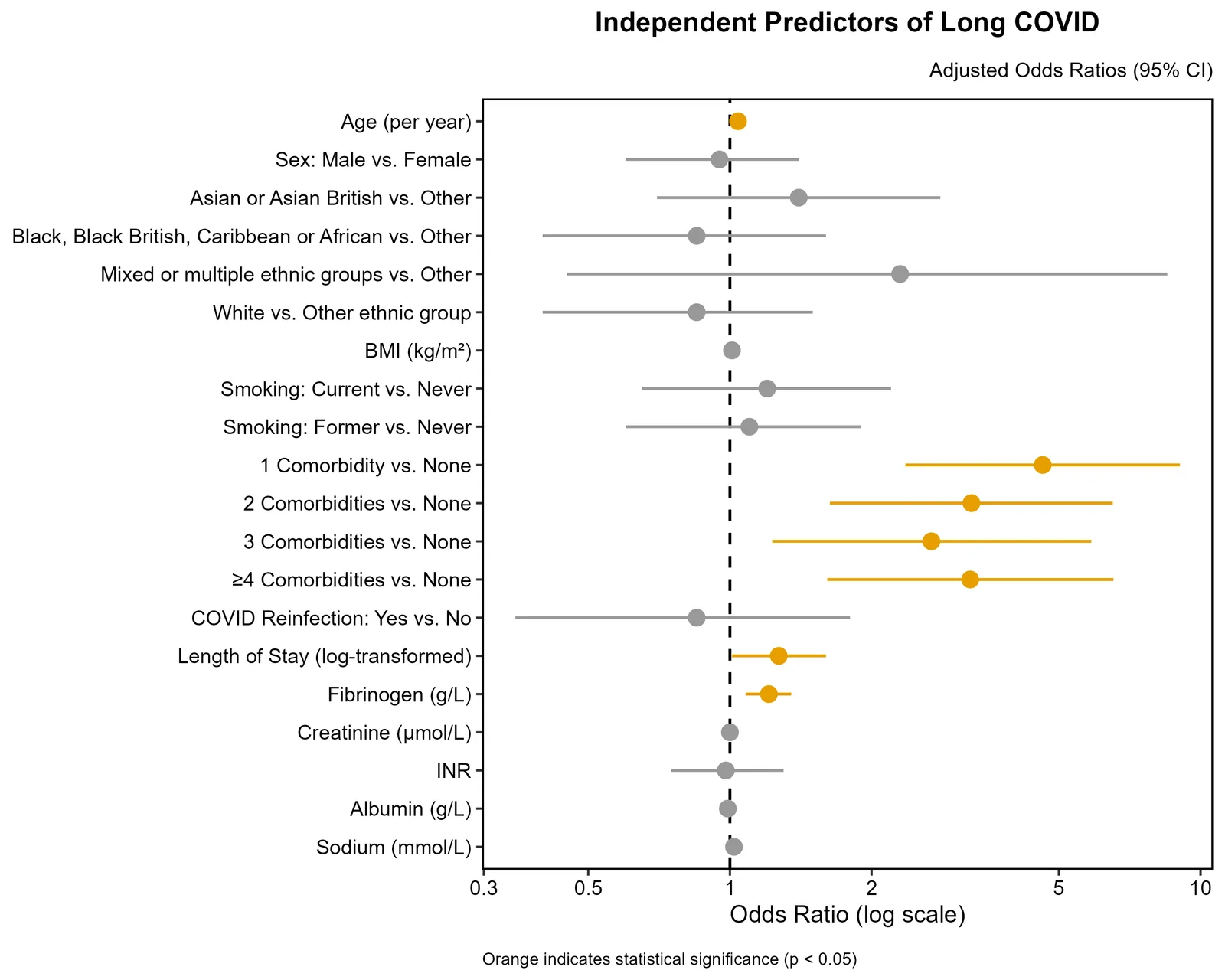
Forest plot of independent predictors of long COVID—visual representation of the multivariable logistic regression analysis presented in Supplementary Table S2. The plot displays adjusted odds ratios (aORs) and 95% confidence intervals. Variables shown in orange were statistically significant (*p* < 0.05), while those in grey were not. The vertical dashed line represents the line of no effect (OR = 1.0). The model was adjusted for age, sex, ethnicity, BMI, smoking status, comorbidities, COVID reinfection, laboratory biomarkers, and acute length of stay.

The multivariable logistic regression analysis identified several independent risk factors for long COVID (Figure 3 and Supplementary Table S2). After adjusting for potential confounders, older age was a significant risk factor; for every one-year increase in age, the odds of developing long COVID increased by 4% (aOR 1.04, 95% CI 1.02–1.05, *p* < 0.001). Biological sex, ethnicity, BMI and smoking were not significantly associated with long COVID. Compared with patients with no underlying health conditions, those with a single comorbidity had a 4.6-fold increase in odds (aOR 4.62, 95% CI 2.36–9.04, *p* < 0.001). The risk remained significantly elevated among patients with 2 (aOR 3.26), 3 (aOR 2.68), or 4 or more conditions (aOR 3.24). Elevated fibrinogen levels at admission were significantly associated with long COVID (aOR 1.21 per g/L, *p* = 0.002). Other laboratory biomarkers—including Creatinine, INR, Albumin, and Sodium—did not show a statistically significant independent association in this model. Acute disease severity, measured by the log-transformed LoS, was an independent predictor (aOR 1.27, *p* = 0.044), indicating that a longer duration of acute hospitalisation was associated with higher odds of persistent symptoms. Neither smoking status (current or former) nor a history of COVID-19 reinfection was a significant predictor of long COVID in this cohort, as indicated by confidence intervals that crossed the no-effect line (OR = 1.0).

### 3.4. Sensitivity Analysis

The robustness of the primary model was evaluated using CCA (Supplementary Figure S4). The independent associations for age and fibrinogen remained statistically significant and similar in magnitude between the imputed and complete-case models, confirming the validity of the multiple imputation strategy. While the point estimates for LoS and lower comorbidity burdens (2–3 conditions) remained directionally consistent with increased risk, their statistical significance was attenuated in the complete-case analysis. This widening of confidence intervals is consistent with the reduction in sample size inherent to complete-case analysis, further justifying the utility of MICE in preserving statistical power. Serum Sodium did not maintain an association in the sensitivity analysis, suggesting it is not a robust predictor.

### 3.5. Phenotypic Evolution of Symptoms

**Figure 4:**
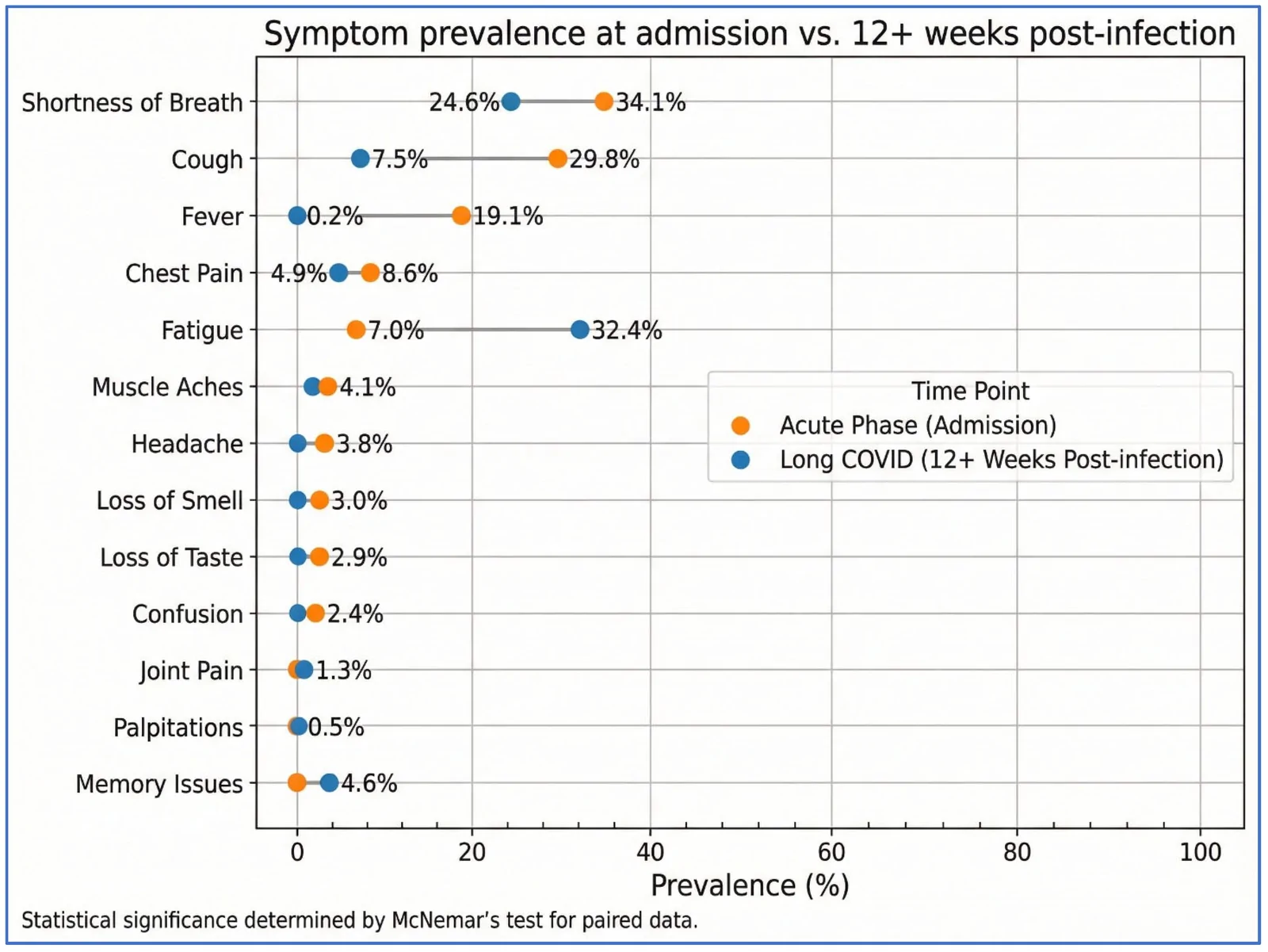
Evolution of symptom prevalence from acute infection to post-acute sequelae. Dumbbell plot illustrating the change in symptom prevalence between hospital admission (orange) and 12+ weeks post-discharge (blue) for matched patients. Numbers represent the percentage of patients reporting the symptom. While typical acute symptoms such as fever and cough showed high rates of resolution, systemic symptoms—specifically fatigue and memory difficulties—exhibited a paradoxical increase in prevalence during the post-acute phase.

The clinical symptom profile underwent a distinct phenotypic shift between the acute and post-acute phases (Figure 4). During the acute infection, the clinical presentation was dominated by respiratory and constitutional symptoms, including shortness of breath (34.1%), cough (29.8%), and fever (19.1%). At the 12-week follow-up, the prevalence of fever and cough declined significantly (*p* < 0.001). However, fatigue emerged as the predominant symptom of long COVID, increasing fourfold from 7.0% at admission to 32.4% at follow-up. Similarly, neurocognitive symptoms, such as memory issues, were more prevalent in the post-acute phase (4.6%) than in the acute phase (0.3%). Shortness of breath persisted for months in nearly a quarter (24.6%) of patients after the initial infection.

### 3.6. Identification of Clinical Phenotypes

**Figure 5:**
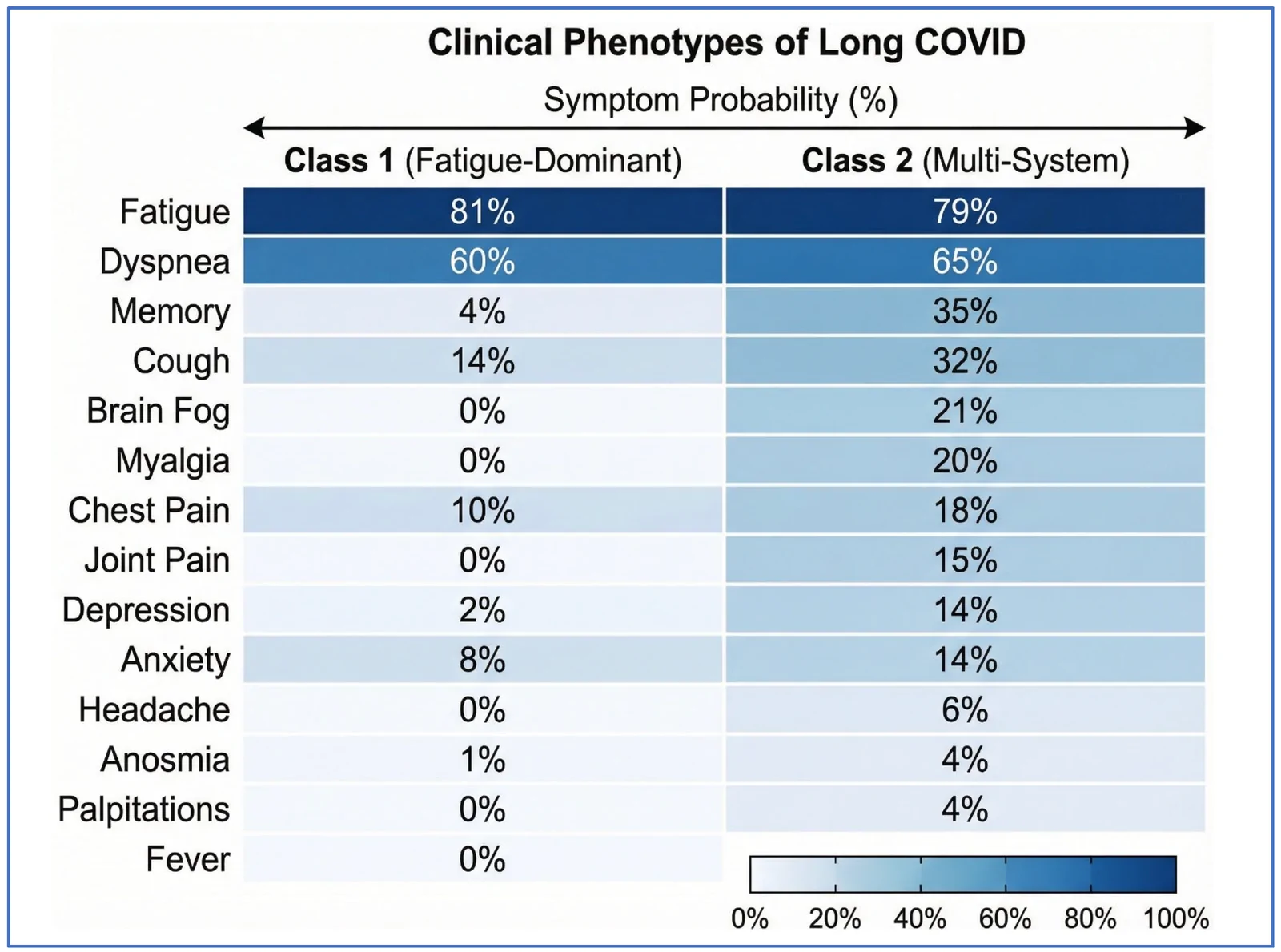
Heatmap of long COVID clinical phenotypes identified via Latent class analysis. The heatmap displays the conditional probability of reporting specific symptoms within the two identified latent classes. Darker blue indicates a higher likelihood of symptom presence; lighter colours indicate low or zero probability. Class 1 (fatigue-dominant) represents a paucisymptomatic phenotype characterised by high probabilities of fatigue (81%) and dyspnoea (60%), but a near-total absence of neurocognitive or musculoskeletal symptoms (0% probability of brain fog, myalgia, or joint pain). A high symptom burden characterises class 2 (multisystem). In addition to the core symptoms of fatigue and dyspnoea, this phenotype exhibits elevated probabilities of neurocognitive dysfunction (memory loss 35%, brain fog 21%), musculoskeletal pain (myalgia 20%, joint pain 15%), and persistent cough (32%).

LCA was performed to identify distinct clinical phenotypes. Models specifying two to four latent classes were fitted and compared. The 2-class solution provided the optimal fit, demonstrating the lowest BIC = 1966.1 compared to the 3-class (BIC = 2017.9) and 4-class (BIC = 2073.1) models (Supplementary Table S3). Consequently, a two-phenotype model was selected for subsequent characterisation and risk factor analysis. LCA identified two distinct clinical phenotypes of long COVID, differentiated by symptom burden and systemic involvement (Figure 5). Fatigue and dyspnoea emerged as the core features of the post-acute condition, presenting with high probability (>60%) across both clusters. However, the clusters were clearly differentiated by the presence of neurocognitive and musculoskeletal symptoms. Class 1: Fatigue-dominant phenotype: This cluster presented with a restricted symptom profile. Although fatigue (81%) and dyspnoea (60%) were highly prevalent, this group reported no cases of brain fog, myalgia, joint pain, headache, or palpitations. Class 2: Multisystem phenotype: This cluster was characterised by a significantly broader symptom burden. In addition to core fatigue and dyspnoea, patients in this group had distinct probabilities of neurocognitive dysfunction (memory difficulties: 35%; brain fog: 21%) and musculoskeletal pain (myalgia: 20%; joint pain: 15%). Furthermore, a persistent cough was more than twice as likely in this group (32%) compared to the fatigue-dominant group (14%).

### 3.7. Predictors of Phenotype Severity

A sub-analysis using logistic regression was performed to identify factors distinguishing the “multisystem” phenotype from the “fatigue-dominant” phenotype (Table 2). Acute disease severity was the strongest predictor of phenotype membership; for every unit increase in the log-transformed length of hospital stay, the odds of developing the multisystem phenotype increased two-fold (OR 2.04, 95% CI 1.23–3.58, *p* = 0.008). Interestingly, an inverse association with age was observed (OR 0.96, *p* = 0.008), indicating that younger patients were significantly more likely to manifest the complex multisystem phenotype, including neurocognitive and musculoskeletal symptoms. In contrast, older patients were more likely to present with the fatigue-dominant phenotype. Admission fibrinogen levels and comorbidity burden did not differ significantly between the two phenotypic clusters, suggesting that these factors drive the overall risk of long COVID rather than the specific symptom presentation.

## 4. Discussion

The research explored the determinants and phenotypic progression of long COVID in hospitalised individuals. Previous research on long-term symptom risks used broad demographics [8,9] but lacked granular biological data due to survey or coding limitations. Prior investigations have predominantly overlooked the integration of acute biomarkers with longitudinal symptom trajectories due to the challenge of missing laboratory data in retrospective cohorts. Consequently, the potential role of specific biological mechanisms, such as coagulopathy, in driving the transition from acute infection to chronic pathology has remained under-explored in multivariable models. This study addresses these gaps by evaluating how acute disease severity, biological markers, and host susceptibility interact to predict long COVID. Our analysis revealed that long COVID following hospitalisation is a predictable outcome driven by a complex interplay of host susceptibility, acute disease severity, and specific biological dysregulation. It is imperative to note that the high prevalence observed in this cohort (40.2%) reflects the severe end of the acute COVID-19 spectrum. These findings cannot be generalised to the broader population of non-hospitalised individuals, who typically experience milder acute infections and exhibit lower overall rates of post-acute sequelae.

We found that the risk of developing post-acute sequelae is heavily stratified by baseline health and the intensity of the initial infection; comorbidity burden emerged as the strongest independent predictor (aOR 4.62), alongside older age and acute severity (measured by length of hospital stay). Also, this study demonstrated a distinct phenotypic shift in disease presentation. Paired symptom analysis showed that, while acute respiratory symptoms such as fever and cough resolved significantly (*p* < 0.001), systemic symptoms, specifically fatigue and neurocognitive dysfunction, paradoxically increased fourfold in the post-acute phase. LCA resolved this heterogeneity into two stable phenotypes: a “fatigue-dominant” cluster and a more severe “multisystem” cluster, with a sub-analysis revealing that younger patients and those with more extended acute hospitalisations were significantly more likely to develop the complex “multisystem” phenotype. The risk of long COVID was not static but evolved with the pandemic; we observed a precipitous decline in incidence from the Wild-type/Alpha waves (>50%) to the Delta/Omicron waves (<21%), highlighting the profound impact of changing viral variants and vaccination on long-term outcomes.

The identification of elevated admission fibrinogen as an independent predictor of long COVID (aOR 1.21) is consistent with the ‘immunothrombosis’ hypothesis [10,24]. However, because other inflammatory markers (such as CRP and ferritin) were excluded due to missingness, fibrinogen likely serves as a broad proxy for the magnitude of the initial systemic inflammatory response. As our retrospective cohort lacks mechanistic assays—such as viral persistence testing, autoantibody screening, or longitudinal microclot detection—we cannot confirm or rule out any specific underlying mechanism. Therefore, these findings suggest an association between acute inflammatory severity and chronic symptoms, rather than definitive mechanistic validation.

The independent significance of acute disease severity, as measured by length of hospital stay, parallels findings from a previous study, which confirmed that the depth of the initial inflammatory insult is a critical driver of post-acute sequelae, separate from baseline host frailty [25].

Rather than a linear dose–response relationship, we observed a strong threshold effect regarding comorbidity burden and long COVID risk. Patients with even a single pre-existing condition exhibited a greater than four-fold increase in odds (aOR 4.62), with the risk remaining elevated but plateauing among those with multiple conditions. This non-monotonic pattern likely reflects the instability of a sparse reference group (only 9.1% of long COVID patients had zero comorbidities) and survival biases inherent in hospitalised populations, highlighting that the presence of any baseline chronic inflammation significantly amplifies the risk of post-acute sequelae. This reinforces the concept of a “syndemic” interaction, in which pre-existing chronic inflammation amplifies viral injury, though the compounding effect of additional comorbidities may be masked by overlapping pathophysiological pathways or survival biases in hospitalised cohorts. While advanced age was a consistent risk factor in our general model, mirroring the findings of a previous study [9], our phenotypic sub-analysis revealed a critical difference; the impact of age is heterogeneous across different clinical presentations.

A novel contribution of this study is the granular characterisation of the disease’s phenotypic evolution. The paradox observed in our paired analysis, where respiratory symptoms resolved while systemic symptoms (fatigue, neurocognitive dysfunction) increased four-fold, challenges the notion of long COVID as merely “lingering” acute disease. Instead, it supports classifying long COVID as a distinct secondary syndrome, likely driven by neuroinflammation or autoimmune mechanisms, as suggested in a previous study [2].

Crucially, our finding that younger patients were significantly more likely to develop the complex multisystem phenotype adds a critical nuance to the existing literature. The precise mechanisms driving this age-related phenotypic divergence remain unclear. We hypothesise that age-related differences in immune mounting—such as the potential for younger patients to trigger a more widespread, yet dysregulated, systemic inflammatory response—could play a role. However, this remains speculative. Future prospective immunological studies are required to elucidate why older patients in our cohort predominantly presented with respiratory and fatigue symptoms, whereas younger patients exhibited broader neurocognitive and musculoskeletal involvement.

Finally, our observation of a precipitous decline in risk from the Wild-type/Alpha waves (>50%) to the Omicron wave (~20%) confirms that the landscape of long COVID is fundamentally changing. This aligns with data from previous studies [25,26], which demonstrated a lower incidence of post-acute sequelae following Omicron infection. This temporal shift likely reflects a combination of the altered intrinsic virulence of newer variants, widespread population immunity derived from national vaccination campaigns and prior infections, and improvements in acute clinical management (e.g., corticosteroids and antivirals). Importantly, our temporal analysis relied on calendar periods as a proxy for variant dominance. This approach carries an inherent risk of exposure misclassification, particularly during transitional periods between waves. Therefore, the observed reduction in long COVID risk cannot be attributed solely to viral mutations but rather to a complex interplay among these confounding temporal factors. However, the persistence of a 20% risk among hospitalised patients during the Omicron era indicates that long COVID remains a significant clinical burden even with milder variants.

Despite the methodological strengths of using multiple imputation and paired within-patient analyses, several limitations must be acknowledged. First, as a single-centre retrospective study focused exclusively on hospitalised patients, our findings reflect local demographics and the presentation of severe illness, limiting generalisability to milder, non-hospitalised cohorts. Furthermore, the observational design precludes causal inference; the association between elevated admission fibrinogen and long COVID reflects correlation rather than causation, as unmeasured confounders such as genetic predisposition or initial viral load could influence both acute severity and long-term outcomes. Statistically, selecting laboratory variables for the multivariable model based on univariate screening (*p* < 0.05) risks inflating apparent statistical significance and potentially omitting complex confounders.

A central limitation of this study is its reliance on EPRs and on retrospective abstraction of clinical notes. The lack of prospective patient-reported outcome measures (PROMs) likely leads to an underestimation of subjective symptoms—such as mild fatigue, anxiety, or brain fog—due to under-documentation by treating clinicians. This reliance on EHRs also introduces significant surveillance (ascertainment) bias. Patients with a high comorbidity burden or severe acute illness (longer LoS) inherently require more frequent post-discharge clinical encounters. This increased healthcare contact time provides these patients with substantially more opportunities for persistent symptoms to be detected and documented than for healthier individuals, who may recover at home without further medical follow-up, potentially inflating the apparent risk associated with multimorbidity. Moreover, while a rigorous adjudication process was utilised to distinguish long COVID from pre-existing comorbidities, retrospective reviews are inherently constrained by the precision of clinician documentation, meaning some symptom misattribution cannot be entirely ruled out.

Additionally, the pandemic’s chronological progression led to inherently uneven follow-up durations across the cohort. Patients infected during early waves had a longer observation window in the EHR to accrue a diagnosis of persistent symptoms, whereas those from later waves (e.g., Omicron) had much shorter follow-up times. This introduces a risk of right-censoring, which may artificially inflate the ‘resolved’ classification in later cohorts. Compounding this, our operational definition of ‘resolved’ equated an absence of documented clinical encounters after 12 weeks with clinical recovery. This approach risks misclassifying patients who were lost to follow-up, sought care outside our hospital network, or experienced symptoms they deemed too mild to report, potentially underestimating the true prevalence of the condition.

Despite these limitations, our study demonstrates that biological markers of coagulation and acute severity are detectable at admission, offering a crucial window for early risk stratification. Future research should prioritise longitudinal, mechanistic studies to investigate whether normalising fibrinogen levels or administering early anticoagulant therapy during the acute phase can mitigate the development of the complex “multisystem” phenotype. Furthermore, the distinct clustering of neurocognitive symptoms in younger patients warrants targeted investigation into neuroinflammatory biomarkers. Given the evolving nature of the virus, continuous epidemiological monitoring is required to determine if the lower risk observed during the Omicron wave persists with future variants or if waning population immunity leads to a resurgence of post-acute sequelae.

## 5. Conclusions

This study highlights long COVID as a heterogeneous syndrome driven by host susceptibility, acute disease severity, and elevated acute inflammatory/coagulation markers. We demonstrated a distinct phenotypic shift in the disease course, evolving from acute respiratory distress to post-acute syndromes dominated by fatigue and neurocognitive dysfunction. The stratification of this condition into distinct ‘fatigue-dominant’ and ‘multisystem’ phenotypes challenges a monolithic view of long COVID. While our findings suggest that a high comorbidity burden and elevated fibrinogen are associated predictors of persistent sequelae, these retrospective observations require validation in large prospective cohorts. Ultimately, understanding these clinical subtypes is a foundational step toward developing targeted, phenotype-specific therapeutic trials for patients suffering from long COVID.

## Figures and Tables

**Table 1:** Baseline characteristics of the study population stratified by long COVID outcome.

Variable	Overall (N = 627)	Resolved (N = 375)	Long COVID (N = 252)	*p*-Value
Age (years)	59 (20)	53 (20)	69 (16)	<0.001 ***
BMI (kg/m^2^)	28 (24, 33)	28 (23, 33)	28 (24, 34)	0.290
LoS (days)	5.0 (3.0, 7.0)	4.0 (3.0, 7.0)	6.0 (4.0, 9.0)	<0.001 ***
Creatinine (µmol/L)	78 (63, 95)	76 (60, 91)	81 (66, 105)	0.001 **
Fibrinogen (g/L)	5.40(4.20, 6.90)	5.20 (3.80, 6.50)	5.70 (4.50, 7.20)	<0.001 ***
INR	1.10 (1.00, 1.10)	1.00 (1.00, 1.10)	1.10 (1.00, 1.20)	<0.001 ***
Albumin (g/L)	32.0 (28.0, 36.0)	33.0 (29.0, 36.0)	31.0 (28.0, 35.0)	0.024*
Sodium (mmol/L)	137.0 (135.0, 140.0)	137.0 (135.0, 139.0)	138.0 (135.0, 140.0)	0.057
Sex				0.269
Female	344 (55%)	213 (57%)	131 (52%)	
Male	283 (45%)	162 (43%)	121 (48%)	
Ethnicity				0.738
Any other ethnic	90 (14%)	57 (15%)	33 (13%)	
Asian/Asian British	118 (19%)	65 (17%)	53 (21%)	
Black, Black British, Caribbean or African	145 (23%)	86 (23%)	59 (23%)	
Mixed/multiple ethnic	18 (2.9%)	12 (3.2%)	6 (2.4%)	
White	256 (41%)	155 (41%)	101 (40%)	
Smoking Status				0.035*
Never smoker	390 (62%)	245 (65%)	145 (58%)	
Unsure	102 (16%)	51 (14%)	51 (20%)	
Current smoker	55 (8.8%)	37 (9.9%)	18 (7.1%)	
Former smoker	80 (13%)	42 (11%)	38 (15%)	
Comorbidity Burden				<0.001 ***
0 conditions	169 (27%)	146 (39%)	23 (9.1%)	
1 condition	95 (15%)	53 (14%)	42 (17%)	
2 conditions	85 (14%)	44 (12%)	41 (16%)	
3 conditions	68 (11%)	37 (9.9%)	31 (12%)	
≥4 conditions	210 (33%)	95 (25%)	115 (46%)	
Vaccination status				
Vaccinated (≥1 dose)	393 (62.7%)	210 (56.0%)	183 (72.6%)	<0.001 ***

BMI = Body Mass Index; length of hospital stay (LoS); INR = International Normalised Ratio; IQR = Interquartile Range; SD = Standard Deviation. Data were presented as mean (SD) for age; median (25th, 75th percentiles) for BMI, length of hospital stay, and laboratory biomarkers; and n(%) for categorical variables. *p*-values were calculated using Welch’s two-sample *t*-test for age; Mann–Whitney U for BMI, length of stay, and laboratory biomarkers; and Pearson’s Chi-squared test for categorical variables. * *p* < 0.05, ** *p* < 0.01, *** *p* < 0.001 indicate statistical significance.

**Table 2:** Multivariable logistic regression identifying predictors of the “multisystem” long COVID phenotype.

Characteristic	OR	95% CI	*p*-Value
Age	0.96	0.93, 0.99	0.008
Sex			
Female	—	—	
Male	0.48	0.21, 1.06	0.075
Comorbidity			
0	—	—	
1	1.25	0.34, 4.95	0.7
2	0.66	0.15, 2.89	0.6
3+	0.76	0.19, 3.34	0.7
Fibrinogen	0.98	0.79, 1.22	0.9
Log LoS	2.04	1.23, 3.58	0.008

The model compares patients in the multisystem cluster (Outcome = 1) against those in the fatigue-dominant cluster (Reference = 0). OR > 1 indicates an increased likelihood of belonging to the multisystem phenotype. Abbreviations: CI = Confidence Interval, OR = Odds Ratio.

## Data Availability

Data will be made available on request. For data requests, kindly contact the corresponding author at Kingston University London via email: k2255680@kingston.ac.uk. The data are not publicly available due to [privacy or ethical restrictions].

## Supplementary Materials

The following supporting information can be downloaded at: https://www.mdpi.com/article/10.3390/biomedicines14081662/s1.
