# Supplementary material for: Phenotypic Evolution, Clinical Subtypes, and Independent Predictors of Long COVID: A Retrospective Cohort Study
Source: Biomedicines. 2026 Jul 24;14(8):1662. doi: 10.3390/biomedicines14081662 (PMC13510062; doi:10.3390/biomedicines14081662)
Supplement: Supplementary file 1 [file biomedicines-14-01662-s001.zip › biomedicines-4410230-supplementary.pdf]

Supplementary Materials

Supplementary Figure S1

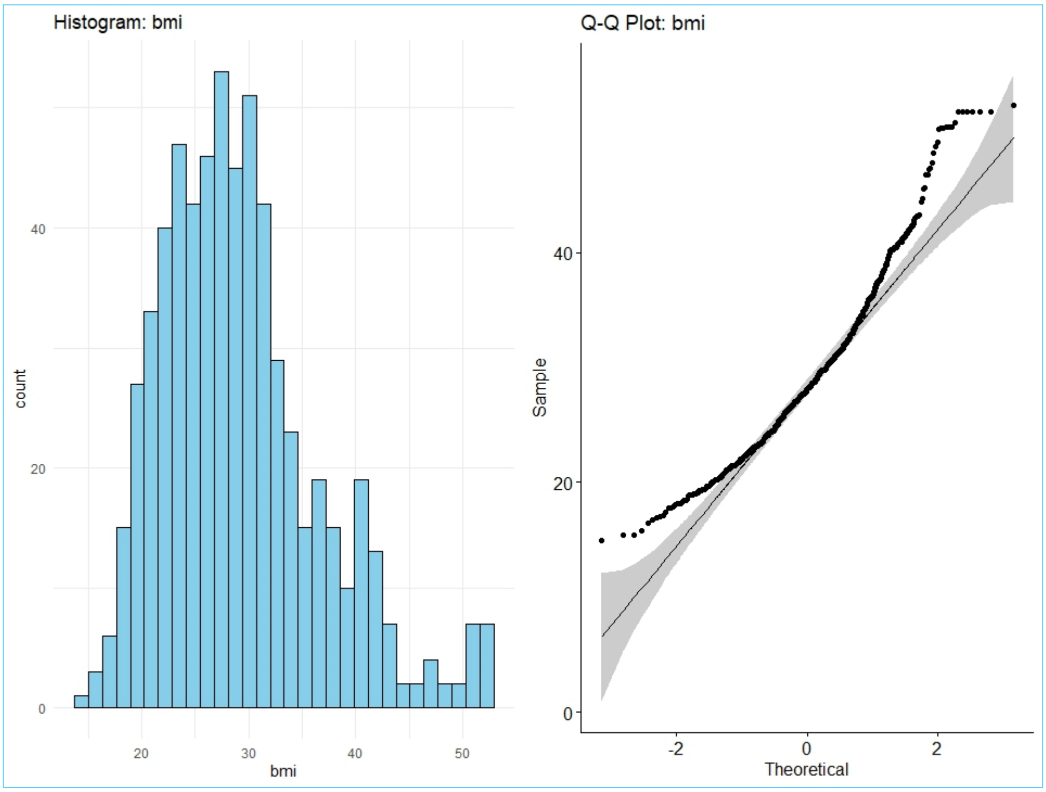

Supplementary Figure S1: Histogram (Left): The shape is not a perfect symmetric bell curve. It has a "Long Tail" to the right (Positive Skew). The bars extend beyond BMI 40 and 50, while the left side stops abruptly around 18. This indicates the presence of outlier patients with very high BMI. Q-Q Plot (Right): The Rule: If the data were normal, the black dots would stay strictly on the straight diagonal line within the grey shaded area. At the top right corner. The black dots curve sharply upwards, away from the line. This confirms that the data are right-skewed (Non-Normal). Decision: Non-Normal. Because of the skew seen in the Q-Q plot, you should treat BMI as a Non-Normal variable. Report as: Median (Interquartile Range - IQR). Test: Mann-Whitney U Test (Wilcoxon rank sum test).

Supplementary Figure S2

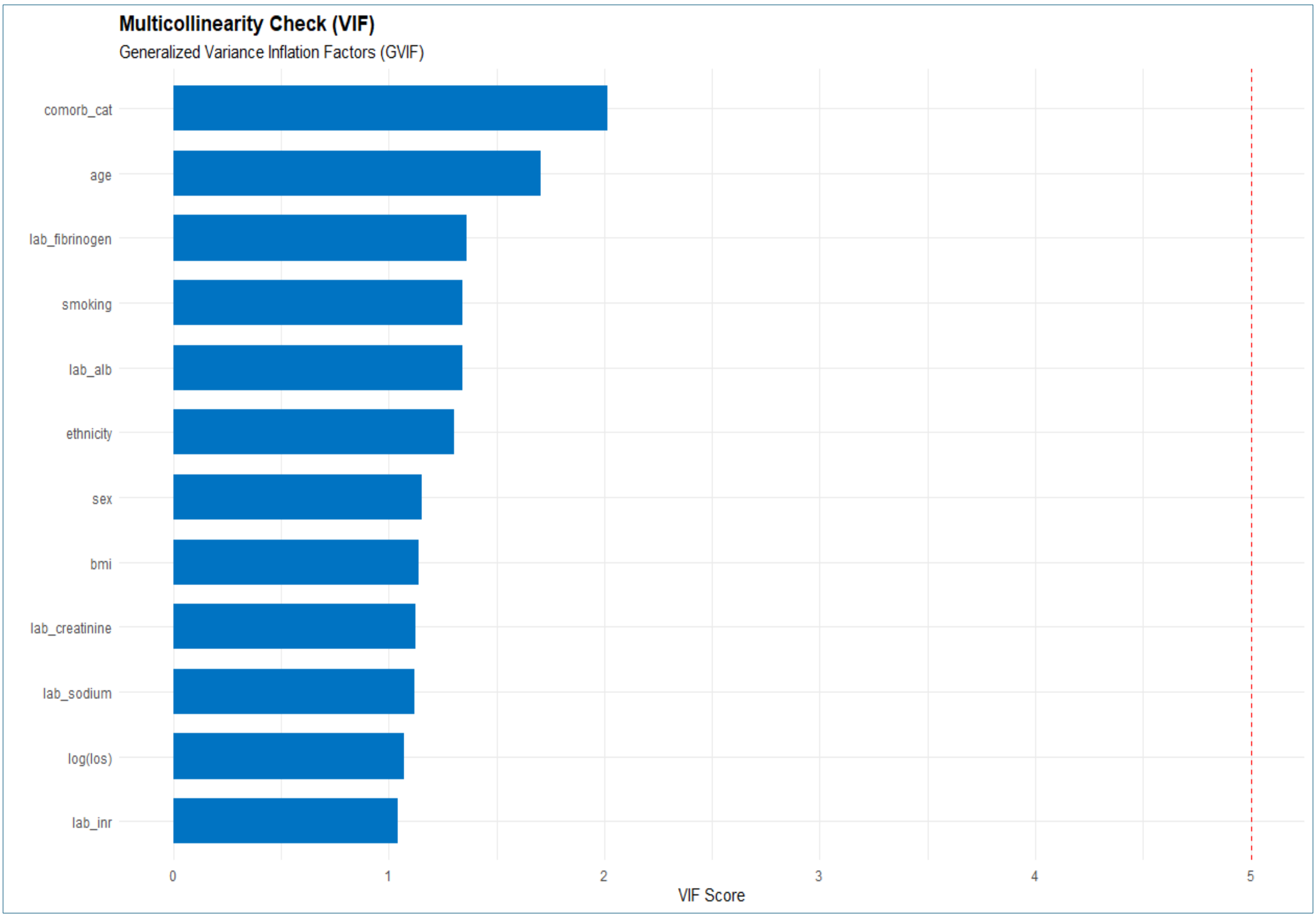

Supplementary Figure S2. Assessment of multicollinearity among predictor variables. Bar chart displaying the Generalised Variance Inflation Factors (GVIF) for all covariates included in the final multivariable logistic regression model. The red dashed line indicates the standard threshold of 5. All variables exhibited GVIF values well below this threshold (maximum GVIF < 2.1), confirming that multicollinearity was not a significant source of bias in the model estimates.

Supplementary Figure S3

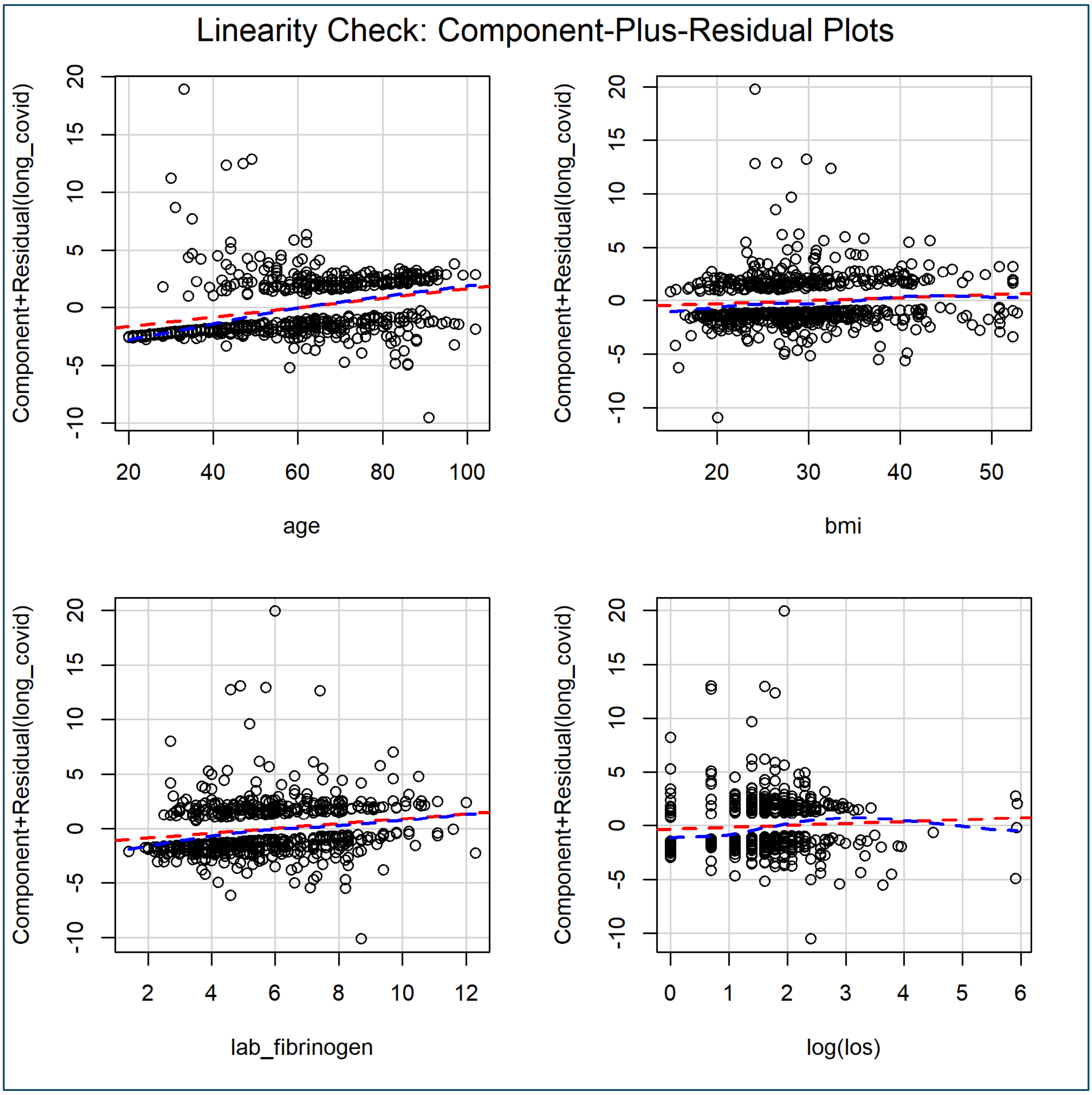

Supplementary Figure S3. Component-plus-residual plots for continuous predictors. Plots assessing the linearity assumption for continuous variables in the multivariable logistic regression model. The red dashed line represents the linear fit modelled by the regression, while the blue line represents the LOESS (locally weighted smoothing) fit to the actual data residuals. (A) Age: Demonstrates a clear linear positive association. (B) BMI: Shows a flat linear relationship, consistent with the non-significant finding in the main model. (C) Fibrinogen: Shows a linear positive trend. (D) Log (Length of Stay): Shows a linear positive association, confirming the appropriateness of the logarithmic transformation for this variable. The close alignment between the linear and smoothed lines across all four plots indicates that the assumption of linearity in the logit scale is satisfied.

Supplementary Figure S4

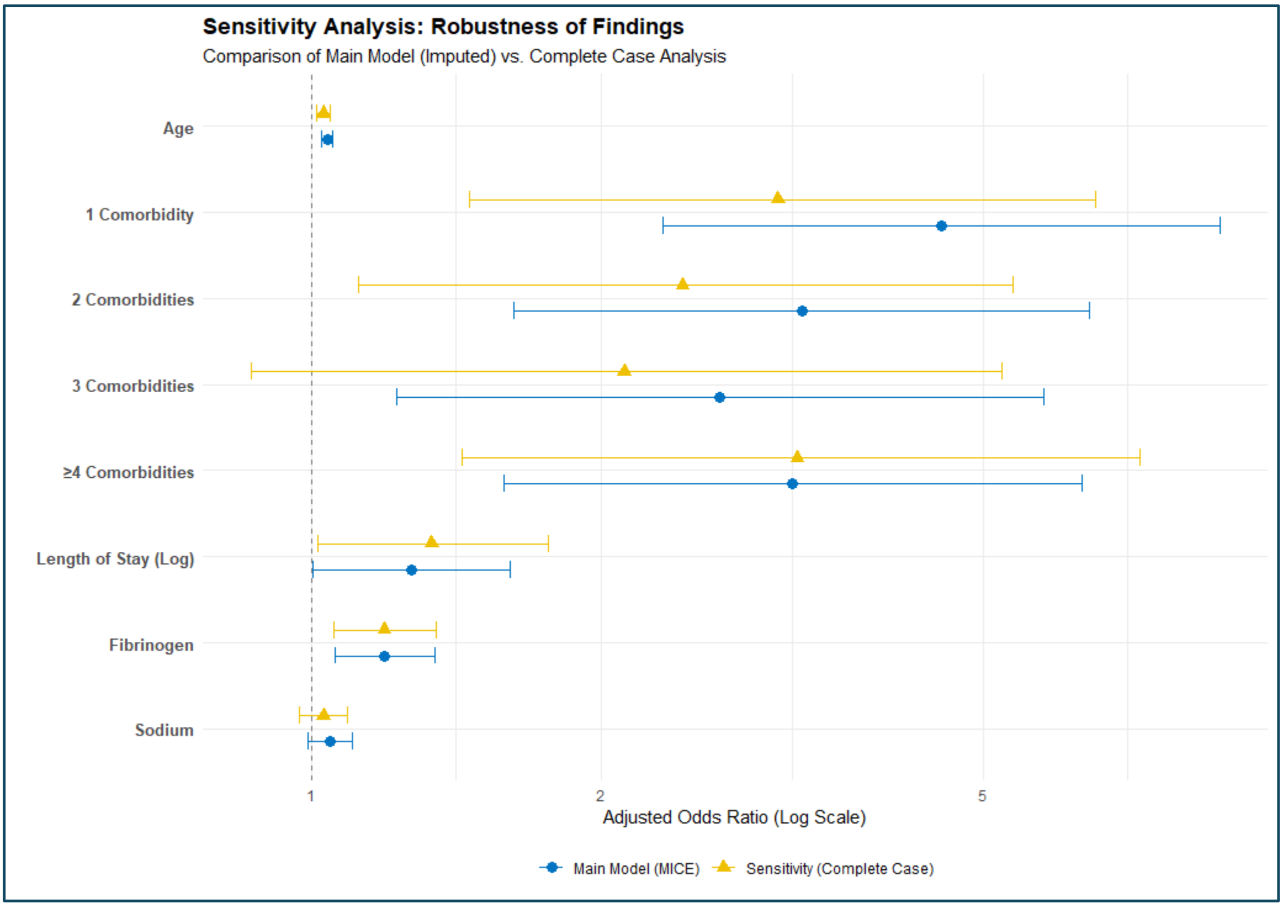

Supplementary Figure S4. Sensitivity analysis assessing the robustness of risk estimates.
Forest plot comparing Adjusted Odds Ratios (aOR) between the primary multivariable model using multiple imputation (Blue circles) and the sensitivity analysis using complete-case data (Yellow triangles). Horizontal lines represent 95% Confidence Intervals.
Interpretation: There is a high degree of concordance between the two models for Age, Fibrinogen, and the highest burden of Comorbidities (≥4), confirming these as robust independent predictors. For Length of Stay and intermediate comorbidity counts (2–3), the complete-case analysis shows wider confidence intervals crossing the line of no effect (OR=1.0); however, the direction and magnitude of the point estimates remain consistent with the primary model, suggesting that the loss of significance in the sensitivity analysis is attributable to reduced statistical power rather than a change in the underlying association.

Supplementary Table S1:Missing Data Analysis for Laboratory Biomarkers at Admission

Laboratory Biomarker	Missing Count (n = 627)	Missing Percentage (%)
Transferrin Binding Capacity	618	98.6
Transferrin	617	98.4
Serum Folate	587	93.6
Vitamin B12	581	92.7
Ferritin	542	86.4
D-dimer	376	60.0
Fibrinogen (Clauss)	159	25.4
Activated Partial Thromboplastin Time (APTT) ratio	154	24.6
International Normalised Ratio (INR)	151	24.1
Mean Corpuscular Volume (MCV)	148	23.6
Monocyte Count	139	22.2
Basophil Count	81	12.9
Eosinophil Count	80	12.8
Mean Corpuscular Haemoglobin Concentration (MCHC)	79	12.6
Red Cell Count (RCC)	78	12.4
Mean Corpuscular Haemoglobin (MCH)	78	12.4
Haematocrit	77	12.3
Red Blood Cell Distribution Width (RDW)	77	12.3
Platelet Count	77	12.3
Neutrophil Count	76	12.1
Lymphocyte Count	74	11.8
Haemoglobin	73	11.6
White Cell Count (WCC)	73	11.6

Summary of missing data frequencies for laboratory biomarkers recorded at the time of acute hospital admission. In accordance with the study's analytical protocol, variables exhibiting >50% missingness (e.g., D-dimer, Ferritin) were excluded from multivariable modelling to prevent imputation bias. Variables with <30% missingness (e.g., Fibrinogen, INR, and full blood count indices) were retained and imputed using Multiple Imputation by Chained Equations (MICE).

Supplementary Table S2: Multivariable logistic regression analysis identifying independent predictors of long COVID

Variable	Adjusted OR (95% CI)	*p*-value
Sociodemographic Factors		
Age (per year)	1.04 (1.02–1.05)	<0.001**
Sex		
Female	Reference	—
Male	0.95 (0.64–1.41)	0.80
Ethnicity		
Any other ethnic group	Reference	—
Asian or Asian British	1.50 (0.78–2.88)	0.20
Black, Black British, Caribbean or African	0.87 (0.46–1.66)	0.70
Mixed or multiple ethnic groups	1.99 (0.52–7.56)	0.30
White	0.84 (0.46–1.51)	0.60
BMI (kg/m^2)	1.02 (1.00–1.05)	0.091
Lifestyle and Clinical History		
Smoking Status		
Never	Reference	—
Current	1.23 (0.60–2.50)	0.60
Former	1.15 (0.65–2.06)	0.60
Comorbidity Burden		
0 conditions	Reference	—
1 condition	4.62 (2.36–9.04)	<0.001**
2 conditions	3.26 (1.63–6.51)	<0.001**
3 conditions	2.68 (1.23–5.86)	0.013**
≥4 conditions	3.24 (1.61–6.53)	0.001**
COVID Reinfection		
No	Reference	—
Yes	0.83 (0.36–1.93)	0.70
Acute Severity & Biomarkers		
Length of Stay (Log-transformed)	1.27 (1.01–1.61)	0.044**
Fibrinogen (g/L)	1.21 (1.07–1.36)	0.002**

Creatinine (μmol/L)	1.00 (1.00–1.00)	0.12
INR	0.99 (0.72–1.36)	>0.90
Albumin (g/L)	0.99 (0.95–1.04)	0.80
Sodium (mmol/L)	1.05 (1.00–1.10)	0.078

Abbreviations: OR = Odds Ratio; CI = Confidence Interval; BMI = Body Mass Index; INR = International Normalised Ratio. Note: ** indicate statistical significance ($p < 0.05$). The model was fit using multiple imputation (m=30) to account for missing laboratory data. Odds ratios were adjusted for all other variables listed in the table.

Supplementary Table S3. Goodness-of-fit statistics for Latent Class Analysis models.

Number of Classes	Log-Likelihood	AIC	BIC
**2**	**-902.86**	**1863.72**	**1966.07**
**3**	-887.30	1862.60	2017.90
**4**	-873.43	1864.86	2073.10

Note: The 2-class model (bolded) was selected as the optimal solution based on the lowest BIC value. Abbreviations: AIC = Akaike Information Criterion; BIC = Bayesian Information Criterion. The optimal number of latent classes was determined by minimising the BIC value, which balances model fit with parsimony. The 2-class model (bolded) was selected as the preferred solution because it had the lowest BIC (1966.07) among the 3-class and 4-class models, indicating the most robust statistical fit to the data.
